# High performance asymmetric supercapacitor based on Cobalt Nickle Iron-layered double hydroxide/carbon nanofibres and activated carbon

**DOI:** 10.1038/s41598-017-04807-1

**Published:** 2017-07-05

**Authors:** Feifei Wang, Shiguo Sun, Yongqian Xu, Ting Wang, Ruijin Yu, Hongjuan Li

**Affiliations:** Shaanxi Key Laboratory of Natural Products & Chemical Biology, School of Chemistry & Pharmacy, Northwest A&F University, Xinong Road 22, Yangling, Shaanxi 712100 P.R. China

## Abstract

A novel Cobalt Nickle Iron-layered double hydroxide/carbon nanofibres (CoNiFe-LDH/CNFs-0.5) composite was successfully fabricated through an easy *in situ* growth approach. The morphology and composition of the obtained materials were systematically investigated. When the two derived materials were used for supercapacitor electrodes, the CoNiFe-LDH/CNFs-0.5 composite displayed high specific surface area (114.2 m^2^ g^−1^), specific capacitance (1203 F g^−1^ at 1 A g^−1^) and rate capability (77.1% from 1 A g^−1^ to 10 A g^−1^), which were considerably higher than those of pure CoNiFe-LDH. Moreover, the specific capacitance of CoNiFe-LDH/CNFs-0.5 composite remained at 94.4% after 1000 cycles at 20 A g^−1^, suggesting excellent long-time cycle life. The asymmetric supercapacitor based on CoNiFe-LDH/CNFs-0.5 as a positive electrode and activated carbon as a negative electrode was manufactured and it exhibited a specific capacitance of 84.9 F g^−1^ at 1 A g^−1^ and a high energy density of 30.2 W h kg^−1^. More importantly, this device showed long-term cycling stability, with 82.7% capacity retention after 2000 cycles at 10 A g^−1^. Thus, this composite with outstanding electrochemical performance could be a promising electrode material for supercapacitors.

## Introduction

In recent years, the increasing energy demand has prompted extensive research on the development of flexible and environmental friendly energy-storage devices^1, 2^. Supercapacitors are attracting considerable attention owing to their excellent pulse charge-discharge time, high power density and long cycle life compared with secondary batteries and conventional capacitors^1–5^. However, supercapacitors usually suffer from lower energy density than conventional capacitors^6^. The energy density is usually limited by specific capacitance and/or the cell voltage. Fortunately, asymmetric supercapacitor (ASC) can make use of the different potential windows of the two electrodes to improve the energy density^7, 8^. More recently, activated carbon (AC) has been considered as promising negative electrode material for ASC due to its high porosity, low cost and other characteristics^9, 10^. Considerable research efforts have been devoted to the various asymmetric capacitor systems, such as AC//CNT/V_2_O_5_ ASC^11^, AC//Ni_3_(VO_4_)_2_ ASC^12^, AC//CoNi-LDH ASC^13^ and AC//NiTe ASC^14^. Electrode materials are some of the most important factors affecting the performance of electrochemical capacitors. Electrode materials for supercapacitors can be generally divided into electrochemical double-layer capacitance (EDLC) materials and pseudocapacitance (PC) materials based on their energy-storage mechanism^2, 3, 15^. Therefore, developing electrode materials with superior electrochemical performance is highly desired.

To date, carbon materials (such as AC, carbon nanotubes^16^, graphene^3^, carbon nanofibres^17^, and others) have been extensively employed as the most preferred electrode materials for EDLC owing to their high power density, large surface area, superior conductivity and long life cycle. Carbon nanofibres (CNFs), which are relatively inexpensive, longer and more edges, defects or strained regions as active sites for electrochemical reaction as compared to carbon nanotubes (CNTs), have been considered as promising electrode materials for supercapacitors because of their extremely high length-to-diameter ratio, superior special surface area, high stiffness and tensile strength^18–22^. However, the low theoretical specific capacitance of CNFs limits their application in supercapacitors^23^.

Layered double hydroxide (LDH) has been intensively researched as the excellent potential pseudocapactive electrode materials because of their high specific capacitance, high redox activity and excellent ion exchange capability^24, 25^. However, for LDH electrode materials, the relatively low conductivity and cycle life has become a major drawback for their application in energy storage. Recently, numerous efforts have been focused on improving the electrochemical performances of LDH by creating nanostructures and hybridising with CNFs. For instance, Liu *et al*. prepared NiAl-LDH/CNFs composite through a hydrothermal process, and the products provided a maximum specific capacitance of 1613 F g^−1^ at 1 A g^−1^ ^3^. Duan *et al*. reported CoMn-LDH/CNFs composite through an *in situ* growth approach, and the products showed a maximum specific capacitance of 1079 F g^−1^ at 2.1 A g^−1^ ^26^. The group found that the specific capacitance of pure LDH can be enhanced via directly mixing CNFs with pure LDH nanosheets. In addition, Fe-based LDH exhibited great prospect on account of their high capacitance, low cost and environmental friendliness^27^. For example, Liu *et al*. found that pure NiFe-LDH can deliver a capacitance of 145 mA h g^−1^ at 5 A g^−1^ through an *in situ* growth approach^28^. Compared with binary-component LDH materials, the ternary-component LDH materials exhibit considerable prospect because of enhanced conductivity and increased electrochemical active sites derived from the incorporation of a third metal cation. Qiu and co-workers reported that the specific capacitance of NiCoAl-LDH/MWCNT composite reached 1035 F g^−1^ at a current density of 1 A g^−1^ ^29^. The group also found that the nanohybrid electrode consisting of NiCoAl-LDH, CNTs and RGO exhibit excellent electrochemical performance with a specific capacitance about 1188 F g^−1^ at a current density of 1 A g^−1^ ^30^. Beyond that, they producted a flexible architecture materials made of NiCoAl-LDH nanoplates coupled with NiCo-carbonate hydroxide nanowires grown *in situ* on graphite paper using a one-step hydrothermal treatment. The hybrid electrode showed an excellent specific capacitance of 1297 F g^−1^ at a current density of 1 A g^−1^ ^31^. The excellent performance of the ternary-component LDH inspired us to investigate more materials for supercapacitors. In our previous report, which provided us with a good idea, we have fabricated pure CoNiFe-LDH successfully, and the morphology and property of the obtained materials were systematically investigated^32^.

Herein, a novel CoNiFe-LDH/CNFs-0.5 composite was developed using an easy *in situ* growth method as high-performance supercapacitors. The as-prepared CoNiFe-LDH/CNFs-0.5 composite displayed enhanced electrochemical performance with large specific capacitance (1203 F g^−1^ at 1 A g^−1^), high rate capability (77.1% from 1 A g^−1^ to 10 A g^−1^) and excellent long time cycle stability (94.4% after 1000 cycles at 20 A g^−1^). The ASC based on CoNiFe-LDH/CNFs-0.5 as a positive electrode and AC as a negative electrode was manufactured and it exhibited a specific capacitance of 84.9 F g^−1^ at 1 A g^−1^ and a high a energy density of 30.2 W h kg^−1^. More importantly, this device showed long-term cycling stability, with 82.7% capacity retention after 2000 cycles.

## Results and Discussion

**Figure 1:**
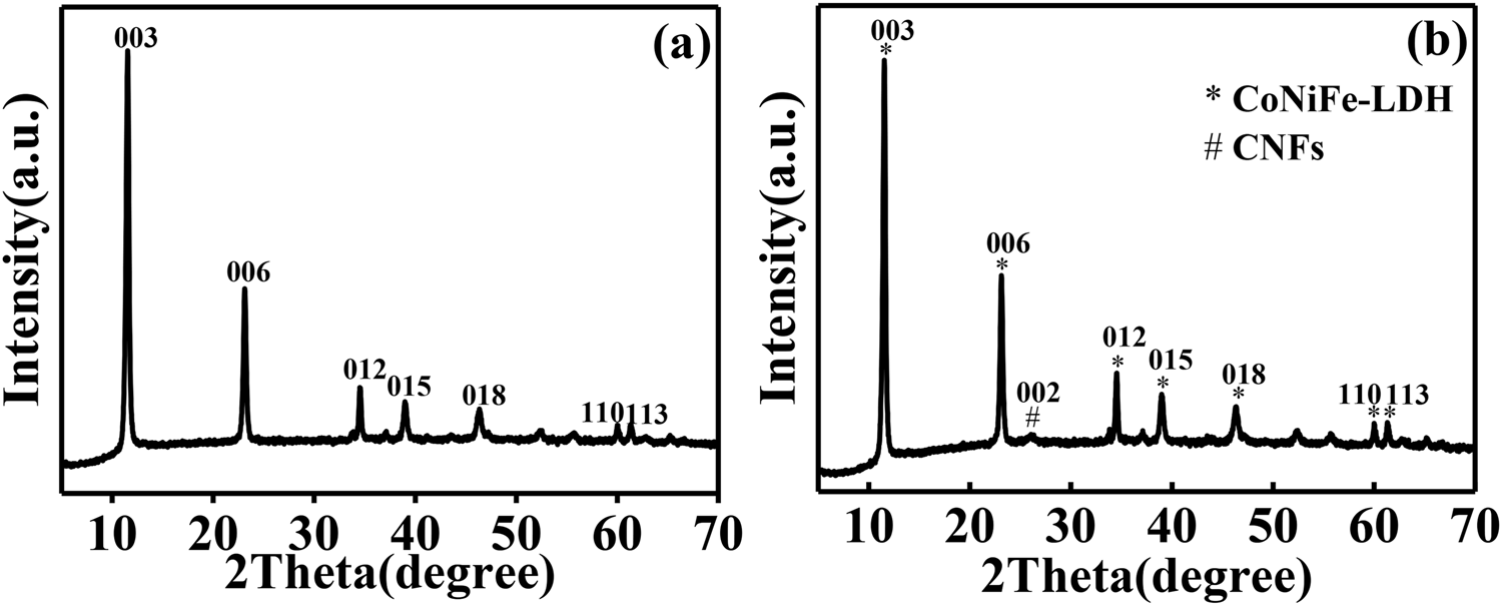
XRD patterns of (**a**) pure CoNiFe-LDH and (**b**) the CoNiFe-LDH/CNFs-0.5 composite.

The XRD patterns of pure CoNiFe-LDH and the CoNiFe-LDH/CNFs-0.5 composite are presented in Fig. 1. For pure CoNiFe-LDH (Fig. 1a), the XRD pattern showed an evident layered structure with a basal spacing of 0.78 nm (2θ = 11.5°). In addition, the characteristic reflections of (003), (006), (012), (015), (018), (110) and (113) can be indexed to a typical hydrotalcite-like structure^33^. In the case of the CoNiFe-LDH/CNFs-0.5 composite (Fig. 1b), the XRD pattern also exhibited hydrotalcite-like characteristics. Moreover, the (002) diffraction peak of CNFs was observed in the CoNiFe-LDH/CNFs-0.5 composite, indicating the existence of CNFs and the formation of the CoNiFe-LDH/CNFs-0.5 composite. (The XRD pattern of CNFs is shown in Fig. S1). The interactions between CoNiFe-LDH and CNFs were further illustrated by Raman spectra as shown in Fig. S2. The Raman shift of CNFs (Fig. S2a) showed the D-band peak at 1346.7 cm^−1^ and G-band peak at 1574.0 cm^−1^, which represented the breathing mode of k-point phonons of A_1g_ symmetry and the first order scattering of the E_2g_ phonons in carbon, respectively^34^. As shown in Fig. S2b, a characteristic peak located at 546.1 cm^−1^ can be observed clearly, which can be related to the CoNiFe-LDH^10^. Besides, Raman spectrum of the CoNiFe-LDH/CNFs-0.5 composite (Fig. S2c) not only displayed typical D band at 1349.4 cm^−1^ and G band at 1578.1 cm^−1^, but also exhibited an obvious band at 544.8 cm^−1^, indicating the successful generation of the CoNiFe-LDH/CNFs-0.5 composite. This was in good agreement with the XRD results.

**Figure 2:**
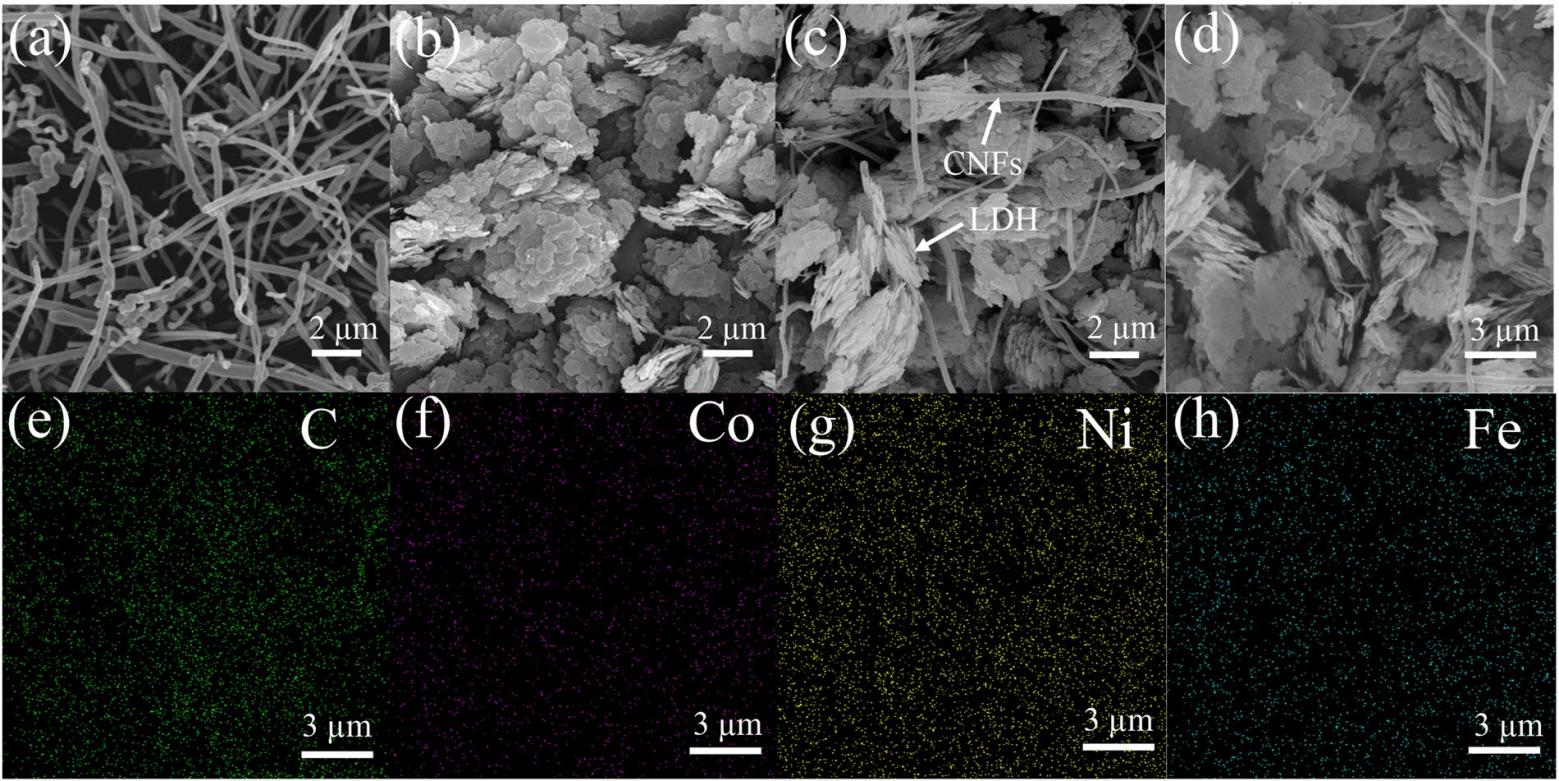
(**a**) SEM image of original CNFs; (**b**) SEM image of the CoNiFe-LDH; (**c**) SEM image of the CoNiFe-LDH/CNFs-0.5 composite; (**d**–**h**) SEM image and the corresponding elemental distribution maps of C, Co, Ni and Fe the CoNiFe-LDH/CNFs-0.5 composite.

In order to inspect the morphology and microstructure of the pristine CNFs, CoNiFe-LDH and the CoNiFe-LDH/CNFs-0.5 composite, the products were characterized by SEM as shown in Fig. 2. The original CNFs (Fig. 2a) were uniformly dispersed and revealed an entangled network. Figure 2b shows the morphology of CoNiFe-LDH. The material consisted of large amount of irregular nanosheets. In Fig. 2c, it can be observed that CNFs were irregular interspersed in the CoNiFe-LDH nanoplatelets. These hierarchically systematic compounded nanostructures were facilitated to debase the aggregation of pure CoNiFe-LDH. Thus, it was advantageous to the ion diffusion and electronic transmission. Furthermore, according to the elemental maps of C, Co, Ni and Fe (Fig. 2(e–h)), it can be found that all elements were uniformly distributed in the CoNiFe-LDH/CNFs-0.5 composite.

**Figure 3:**
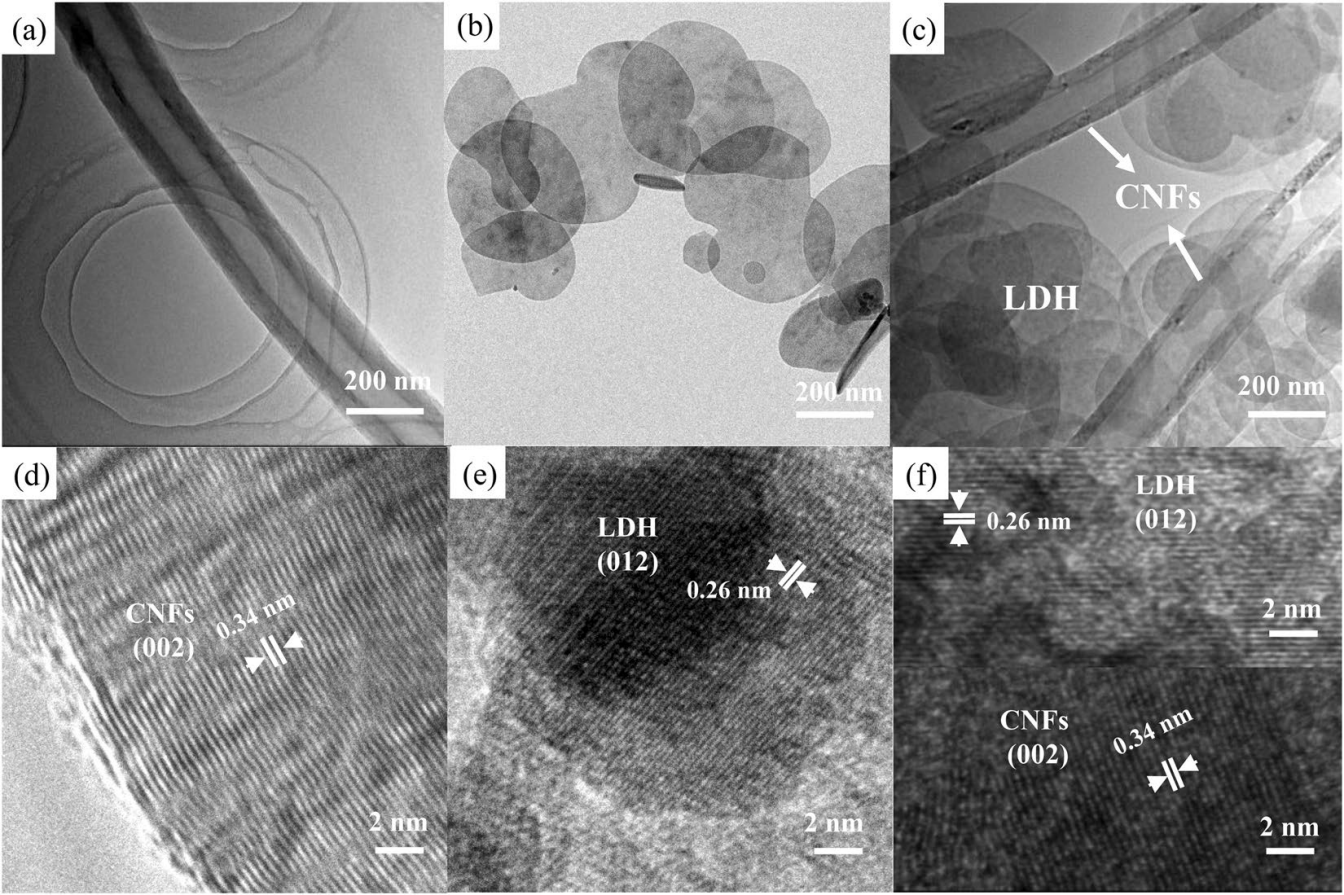
(**a**) TEM image of original CNFs; (**b**) TEM image of the CoNiFe-LDH; (**c**) TEM image of the CoNiFe-LDH/CNFs-0.5 composite; (**d**) HRTEM image of the original CNFs; (**e**) HRTEM image of the CoNiFe-LDH and (**f**) HRTEM image of the CoNiFe-LDH/CNFs-0.5 composite.

To further inspect the morphology and microstructure of pristine CNFs, pure CoNiFe-LDH and the CoNiFe-LDH/CNFs-0.5 composite, the as-prepared samples were characterised by TEM. We can observe that the original CNFs (Fig. 3a) exhibited a typical fibrous morphology with a hollow tubular structure. The inner diameter of CNFs was estimated to be about 100 nm. Pure CoNiFe-LDH showed an irregular platelet-like morphology (Fig. 3b). In Fig. 3c, CNFs were observed to be irregularly interspersed in platelet-like CoNiFe-LDH flakes to form the CoNiFe-LDH/CNFs-0.5 composite. The high-resolution TEM (HRTEM) images of the original CNFs, CoNiFe-LDH and the CoNiFe-LDH/CNFs-0.5 composite are shown in Fig. 3(d–f). The lattice fringe of 0.34 nm was assigned to the (002) lattice plane of CNFs^3^. The inter-planar spacing of 0.26 nm belonged to the (012) lattice plane of CoNiFe-LDH^29^. The CoNiFe-LDH/CNFs-0.5 composite revealed two kinds of contrast fringes, confirming the successful preparation of the compound. This finding was consistent with those of XRD analysis.

**Figure 4:**
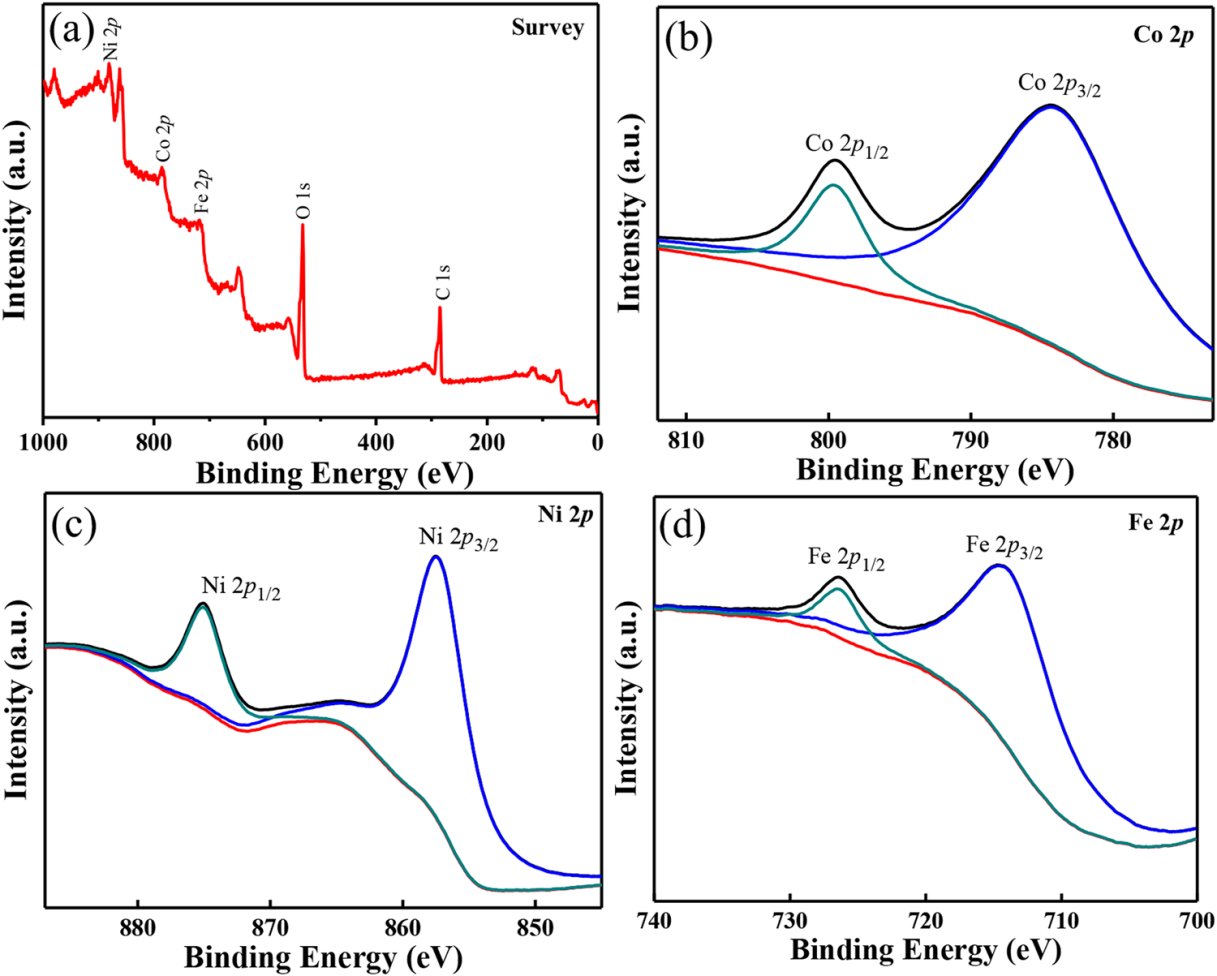
XPS spectra of the CoNiFe-LDH/CNFs-0.5 composite: (**a**) survey spectrum; (**b**) Co 2*p*; (**c**) Ni 2*p* and (**d**) Fe 2*p*.

To obtain more information regarding chemical composition and surface chemical state of the CoNiFe-LDH/CNFs-0.5 composite, we investigated the composite by using X-ray photoelectron spectroscopy (XPS) as shown in Fig. 4. As can be seen from the XPS scan spectrum (Fig. 4a), the CoNiFe-LDH/CNFs-0.5 composite was primarily composed of Co, Ni, Fe, C and O elements. The Co 2*p* XPS spectrum (Fig. 4b) showed two major peaks at 797.6 (Co 2*p*_1/2_) and 782.5 eV (Co 2*p*_3/2_), indicating the Co(II) oxidation state in the composite^3^. For the Ni 2*p* XPS spectrum as shown in Fig. 4c, two major peaks around 874.4 and 856.8 eV corresponded to Ni 2*p*_1/2_ and Ni 2*p*_3/2_, respectively. The spin-energy separation of 17.6 eV is a feature of Ni^2+^, which is consistent with the results reported in the previous literature^3, 35^. The Fe 2*p* XPS spectrum (Fig. 4d) exhibited two peaks located at 714.2 (Fe 2*p*_3/2_) and 726.5 eV (Fe 2*p*_1/2_), suggesting the presence of Fe(III) in the CoNiFe-LDH/CNFs-0.5 composite^36^.

**Figure 5:**
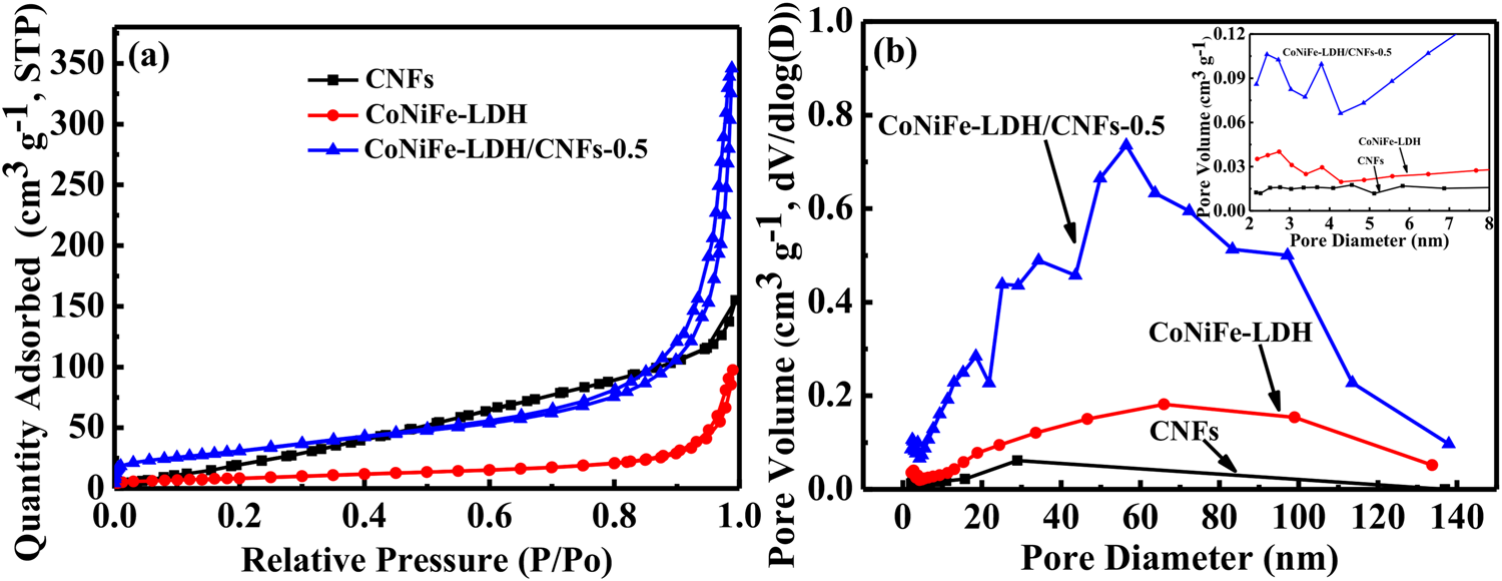
(**a**) Nitrogen adsorption/desorption isotherms of original CNFs, pure CoNiFe-LDH and CoNiFe-LDH/CNFs-0.5 composite; (**b**) the pore size distribution of original CNFs, pure CoNiFe-LDH and CoNiFe-LDH/CNFs-0.5 composite (the inset exhibits the corresponding magnified pore size distribution).

The N_2_ adsorption/desorption technique was used to measure the pore structure of original CNFs, pure CoNiFe-LDH and the CoNiFe-LDH/CNFs-0.5 composite. As shown in Fig. 5a, the specific surface area of the CoNiFe-LDH/CNFs-0.5 composite (114.2 m^2^ g^−1^) was higher than that of original CNFs (88.9 m^2^ g^−1^) and pure CoNiFe-LDH (32.1 m^2^ g^−1^), which may result in superior electrochemical characteristics^37^. This finding proved that the addition of CNFs can decrease the aggregation of pure CoNiFe-LDH, resulting in an enlarged specific surface area^37^. The pore size distribution of original CNFs, pure CoNiFe-LDH and the CoNiFe-LDH/CNFs-0.5 composite is shown in Fig. 5b. As shown in Fig. 5b, the macropore volume of the CoNiFe-LDH/CNFs-0.5 composite between 50 and 100 nm was larger than pure LDH and original CNFs, as ascribed to the abundant interstitial spaces between LDH sheets and CNFs^6^. Reportedly, the pore size distribution within 2–5 nm is optimal for the behaviour of supercapacitors^38^. The pore distribution of pure CoNiFe-LDH and the CoNiFe-LDH/CNFs-0.5 composite exhibited a narrow distribution at 2–4 nm (inset in Fig. 5b), which can be assigned to the mesopores in the LDH sheets. Moreover, the pore volume of the CoNiFe-LDH/CNFs-0.5 composite was larger than that of LDH because the introduction of CNFs will promote the crystallisation of CoNiFe-LDH sheets with a smaller size. The pore distribution and large specific surface area of CoNiFe-LDH/CNFs-0.5 composite will provide efficient transport pathways to their interior voids, which was favourable for improving both the PC from LDH and the EDLC from CNFs^6, 39^.

In order to optimize the electrochemical performance of the electrode, Cyclic voltammetry (CV) and galvanostatic charge-discharge (GCD) of the electrodes with different CNFs concent were investigated using a three-electrode system (Fig. S3). It is clearly seen that the specific capacitance of the CoNiFe-LDH/CNFs-0.5 electrode (1203 F g^−1^) was larger than CoNiFe-LDH/CNFs-0.25 (993 F g^−1^) and CoNiFe-LDH/CNFs-0.75 (1130 F g^−1^). Therefore, 0.5 mg mL^−1^ was chosen as the optimum concentration for further evaluation of its electrochemical performance.

**Figure 6:**
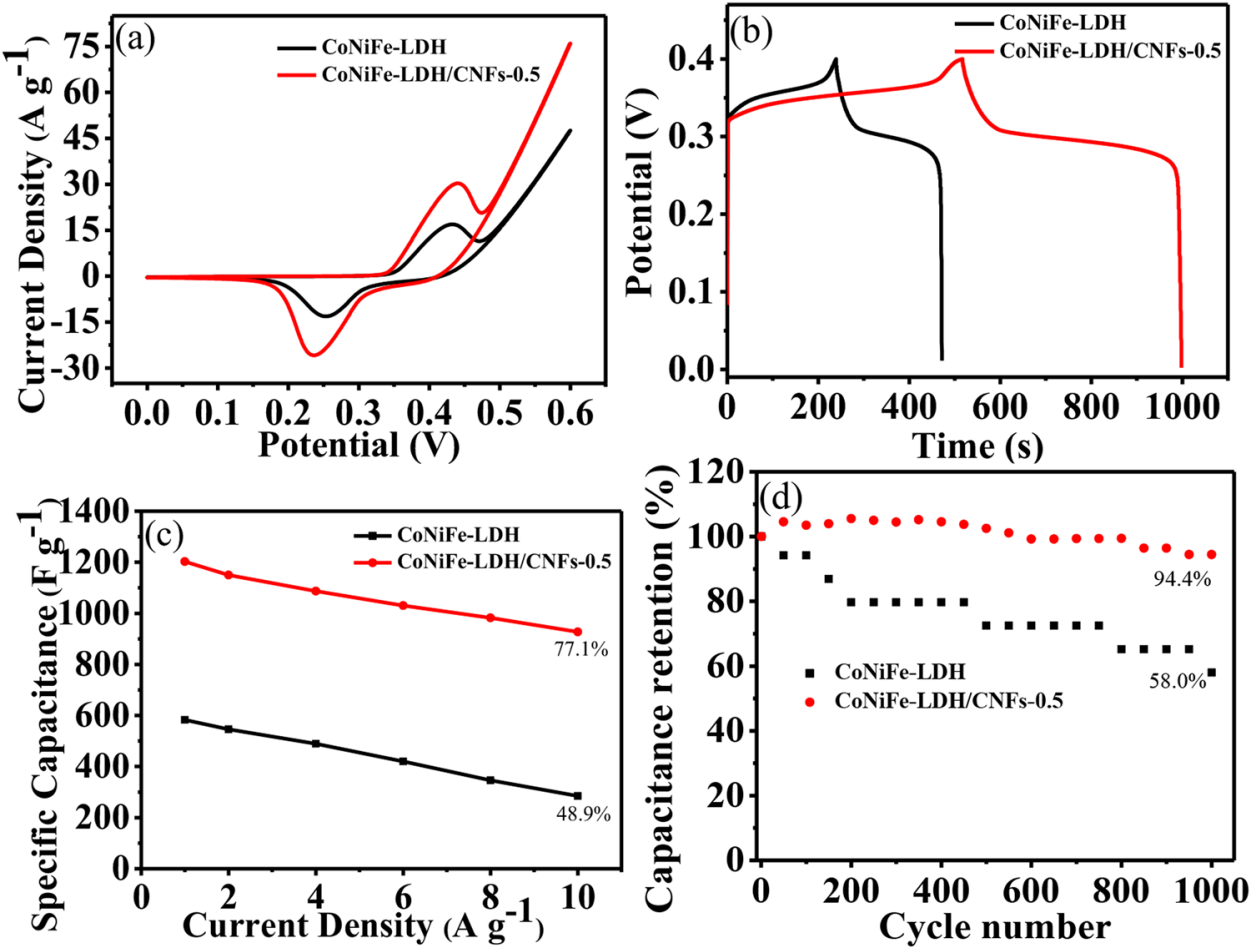
Electrochemical performance of the samples measured in 6 M KOH solution. (**a**) CV curves of pure CoNiFe-LDH and the CoNiFe-LDH/CNFs-0.5 composite at a scan rate of 5 mV s^−1^; (**b**) GCD curves of pure CoNiFe-LDH and the CoNiFe-LDH/CNFs-0.5 composite at a current density of 1 A g^−1^; (**c**) The specific capacitance of pure CoNiFe-LDH and the CoNiFe-LDH/CNFs-0.5 composite at different current densities; (**d**) The cycling performance of the CoNiFe-LDH/CNFs-0.5 composite at a current density of 20 A g^−1^ and the cycling performance of the CoNiFe-LDH at a current density of 10 A g^−1^.

The capacitive performances of pure CoNiFe-LDH and the CoNiFe-LDH/CNFs-0.5 composite were investigated by CV in 6 M KOH solution. Figure 6a shows the CV curves of pure CoNiFe-LDH and the CoNiFe-LDH/CNFs-0.5 composite at a scan rate of 5 mV s^−1^. Evidently, the CV curves of the samples all exhibited a pair of redox peaks, indicating the PC characteristics of the materials. Redox peaks can be attributed to the conversion between the different oxidation states of Ni and Co in the materials. We could see that the area surrounded by CV curves for the CoNiFe-LDH/CNFs-0.5 composite electrode was apparently larger than that of pure CoNiFe-LDH electrode at the scan rate of 5 mV s^−1^, which resulted from the higher electrochemical activity of the CoNiFe-LDH/CNFs-0.5 composite. The CV curves of pure CoNiFe-LDH and the CoNiFe-LDH/CNFs-0.5 composite at different scan rates ranging from 5 to 50 mV s^−1^ are presented in Fig. S4a and b, respectively. With the increase in scan rate, the redox peak current density increased significantly, manifesting faster redox rate on the electrode^3^. The potential difference between anodic and cathodic peaks was used as a measure of the reversibility of the electrochemical redox reaction: the higher the reversibility, the smaller the potential difference^40^. With the increase in scan rate, an increase in potential difference was observed, implying low reversibility at a high scan rate. This finding was mainly on account of the resistance and polarisation of the electrode material^41, 42^.

To further investigate the electrochemical behaviours of electrode materials, we also conducted GCD tests of the samples. Figure 6b shows the GCD curves of pure CoNiFe-LDH and the CoNiFe-LDH/CNFs-0.5 composite at a current density of 1 A g^−1^. According to the charge/discharge curves, the specific capacitance of the CoNiFe-LDH/CNFs-0.5 composite was 1203 F g^−1^ at a current density of 1 A g^−1^, which was much higher than pure CoNiFe-LDH (583 F g^−1^). This finding signified that the unexceptionable electric conductivity of CNFs was beneficial to improve the electroactive surface area and the charge transport on the electrode^43^. The initial charge/discharge measurements of pure CoNiFe-LDH and the CoNiFe-LDH/CNFs-0.5 composite at different current densities are shown in Fig. S5a and b. All curves appear nearly symmetric, which was indicative of excellent electrochemical reversibility^44^. Notably, the capacitance of the CoNiFe-LDH/CNFs-0.5 composite can achieve 500 F g^−1^ at a current density of 20 A g^−1^, whereas pure CoNiFe-LDH can achieve 285 F g^−1^ at a current density of 10 A g^−1^. The result indicated that CNFs can enhance the capacitive behaviour of the CoNiFe-LDH electrode. The specific capacitance of pure CoNiFe-LDH and the CoNiFe-LDH/CNFs-0.5 composite at different current densities is shown in Fig. 6c. When the current density was 1, 2, 4, 6, 8 and 10 A g^−1^, the corresponding specific capacitances of the CoNiFe-LDH/CNFs-0.5 composite were 1203, 1150, 1087, 1030, 982 and 927 F g^−1^, respectively, which were considerably higher than those of pure CoNiFe-LDH (583, 546, 489, 420, 346 and 285 F g^−1^). Notably, the capacitance retention of the CoNiFe-LDH/CNFs-0.5 composite remained at 77.1% when the current density increased from 1 A g^−1^ to 10 A g^−1^, and this value was considerably higher than that of pure CoNiFe-LDH (48.9%). The decrease in the capacitance was due to insufficient time for the electrolyte ions to diffuse and come in good contact with active surfaces at high current density^45^. The result indicated that the incorporation of CNFs into CoNiFe-LDH can significantly improve the specific capacitance and rate capability of the composite, which was consistent with CV results.

Cycling stability is an important parameter in evaluating electrode materials. The cyclic performance of the CoNiFe-LDH/CNFs-0.5 composite electrode was examined by galvanostatic charge/discharge test for 1000 cycles at a current density of 20 A g^−1^. As shown in Fig. 6d, the capacitive retention rate of the CoNiFe-LDH/CNFs-0.5 composite was calculated to be 94.4% after 1000 cycles, which was higher than that of pure CoNiFe-LDH (58.0%), demonstrating the excellent long-time cycle stability.

**Figure 7:**
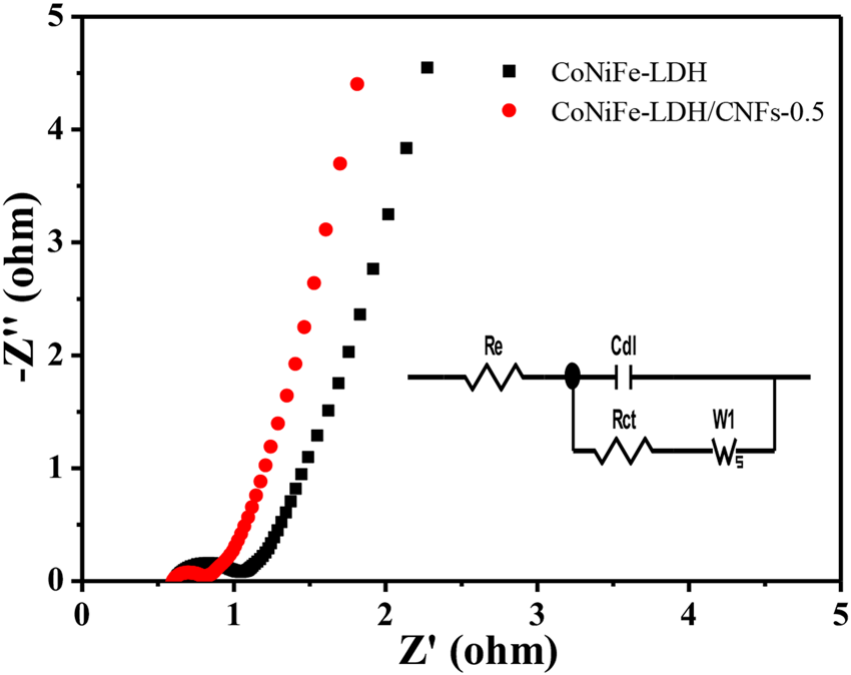
The Nyquist plots of pure CoNiFe-LDH and the CoNiFe-LDH/CNFs-0.5 composite, the inset shows the equivalent circuit diagram.

EIS was also employed to further study the impedance properties of the as-prepared samples. Figure 7 shows the Nyquist plots of pure CoNiFe-LDH and the CoNiFe-LDH/CNFs-0.5 composite. Evidently, the EIS curves of the as-prepared samples were virtually similar in shape, which was composed of an irregular semicircle in the high frequency region and a straight line in the low frequency region. The diameter of the semicircle reflected the charge-transfer resistance (R_ct_) process on the surfaces of electroactive materials and was caused by Faradic reactions^45, 46^. The straight line corresponds to Warburg impedance, which was related to the diffusion of the electroactive species, indicating ion diffusion resistance^47–50^. As shown in Fig. 7, the Nyquist plots of CoNiFe-LDH/CNFs-0.5 composite showed a considerably smaller semicircle than that of pure CoNiFe-LDH, indicating that the CoNiFe-LDH/CNFs-0.5 composite possessed lower charge transfer resistance and higher electron transport. Both CoNiFe-LDH/CNFs-0.5 composite and pure CoNiFe-LDH presented a high slope, suggesting lower diffusion resistance and superior electronic transport of both materials. The EIS pattern can be fitted by an equivalent circuit as shown in the inset of Fig. 7, where R_e_ was the bulk solution resistance, C_dl_ represented the constant phase element accounting for a double-layer capacitance and W_1_ was the Warburg resistance^42, 51^. The calculated R_ct_ of CoNiFe-LDH/CNFs-0.5 was 0.1455 Ω, which was smaller than that of pure CoNiFe-LDH (0.3014 Ω). All results demonstrateed that the addition of CNFs in pure CoNiFe-LDH produced a positive effect on the composite. It not only alleviated the agglomeration of CoNiFe-LDH but also enhanced the electrical conductivity of the composite.

**Figure 8:**
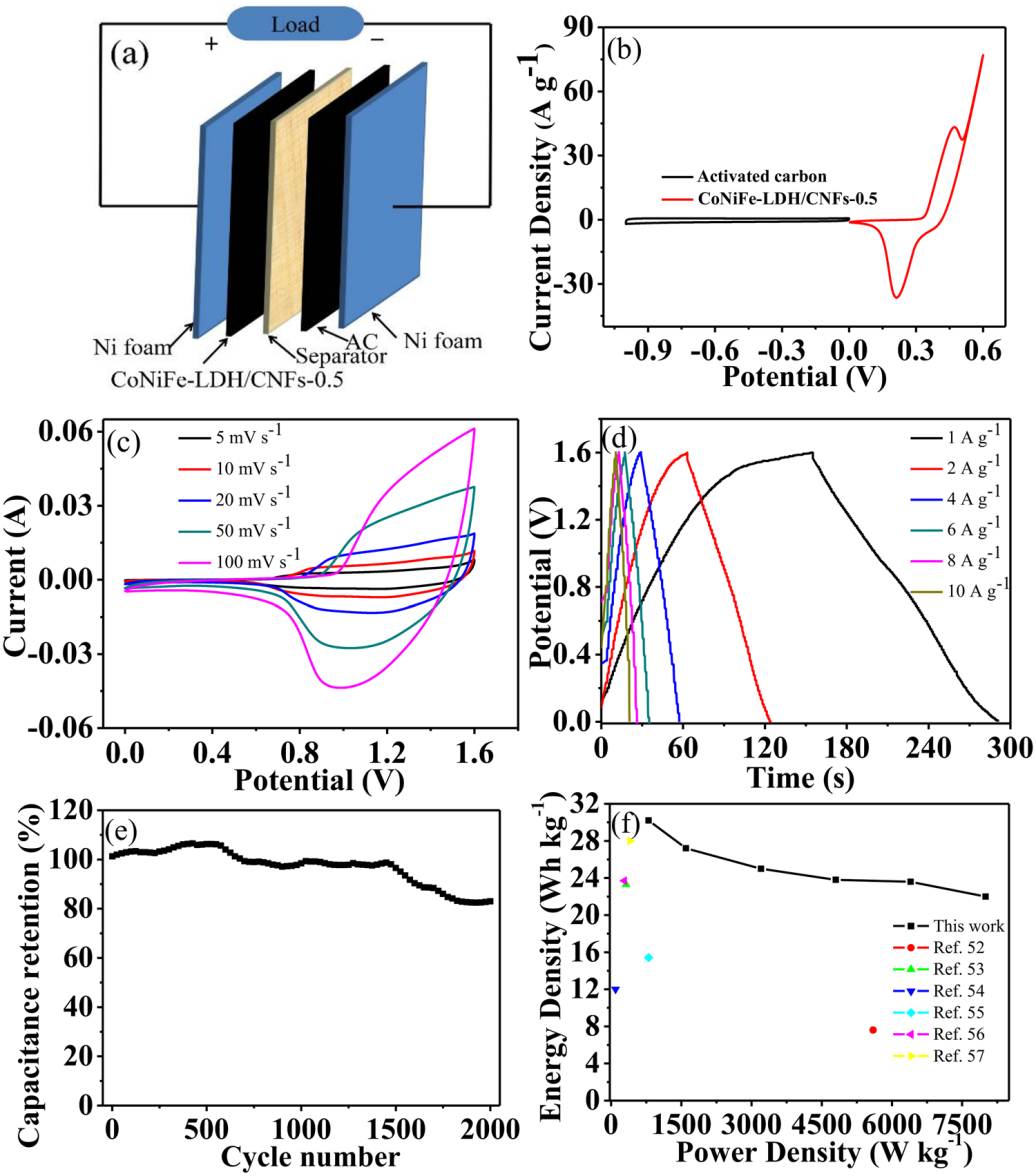
(**a**) The schematic of the AC//CoNiFe-LDH/CNFs-0.5 ASC. (**b**) CV curves of AC and CoNiFe-LDH/CNFs-0.5 electrode at scan rate of 10 mV s^−1^. (**c**) CV curves of the AC//CoNiFe-LDH/CNFs-0.5 ASC at various scan rates. (**d**) The galvanostatic charge-discharge curves of the AC//CoNiFe-LDH/CNFs-0.5 ASC at various current densities. (**e**) Cycling performance of the AC//CoNiFe-LDH/CNFs-0.5 ASC at the current density of 10 A g^−1^. (**f**) Ragone plot of the AC//CoNiFe-LDH/CNFs-0.5 ASC.

Considering the wonderful performance of the CoNiFe-LDH/CNFs-0.5 composites, the AC//CoNiFe-LDH/CNFs-0.5 ASC was fabricated. The schematic diagram of the AC//CoNiFe-LDH/CNFs-0.5 ASC is shown in Fig. 8a. The comparative CV curves of the posotive and negative electrodes measured at scan rate of 10 mV s^−1^ in a three-electrode system is demonstrated in Fig. 8b. It was used to estimate the potential window of the hybrid supercapacitor. By combining the potential window of the CoNiFe-LDH/CNFs-0.5 composites and AC, the AC//CoNiFe-LDH/CNFs-0.5 ASC can achieve a maximum potential of 1.6 V. Figure 8c shows the CV curves of the AC//CoNiFe-LDH/CNFs-0.5 ASC at various scan rates with a potential window of 0 to 1.6 V. We can see that increasing the scan rate leaded to further augmentation of the CV curve, and the result was similar to the CV curves showed in the three-electrode configuration. As observed, the AC//CoNiFe-LDH/CNFs-0.5 ASC exhibited both electrical double-layer capacitor and Faradaic pseudocapacitance capacitor properties at all scan rates. The GCD curves of AC//CoNiFe-LDH/CNFs-0.5 ASC at different current densities are shown in Fig. 8d. Based on the results of discharge curves, the ASC achieved a high specific capacitance of 84.9 F g^−1^ at 1 A g^−1^. Meanwhile, the specific capacitance was 76.5, 70.2, 66.9, 66.5, and 62.0 F g^−1^ at 2, 4, 6, 8 and 10 A g^−1^, respectively, thereby implying good rate capability. Importantly, the AC//CoNiFe-LDH/CNFs-0.5 ASC exhibited wonderful cycling stability with 82.7% specific capacitance retention after 2000 cycles at 10 A g^−1^ (Fig. 8e). In this work, the AC//CoNiFe-LDH/CNFs-0.5 ASC device can display a high energy density of 30.2 W h kg^−1^ at 1 A g^−1^, and the corresponding power density was 800.1 W kg^−1^. The results showed that the AC//CoNiFe-LDH/CNFs-0.5 ASC achieved higher energy density than the reported devices such as AC//NiCoO_x_-GNS ASC (7.6 W h kg^−1^)^52^, AC//NiCo_2_O_4_-rGO ASC (23.3 W h kg^−1^)^53^, AC//NiCoO_x_ ASC (12 W h kg^−1^)^54^, AC//NiCo_2_O_4_ ASC (15.4 W h kg^−1^)^55^, AC//NiCoLDH-Zn_2_SnO_4_ ASC (23.7 W h kg^−1^)^56^, AC//NiCo_2_O_4_@MnO_2_-NF ASC (28 W h kg^−1^)^57^.

## Methods

### Materials

All the chemicals were of analytical grade and used without further purification. Carbon nanofibres (CNFs), with inner diameter of 100 nm, were purchased from Sigma-Aldrich.

### Modification of CNFs

Negatively charged CNFs (CNFs-COOH) were prepared according to methods in the literature^3^. The pristine CNFs were dispersed in concentrated nitric acid (68 wt.%) by ultrasonication for 30 min. Then, the CNFs suspension was refluxed at 100 °C for 12 h. A large number of carboxyl groups (COOH) have been generated on the surface of CNFs after nitric acid treatment. The resulting acid-treated CNFs were separated by filtration, washed with deionised water and dried at 80 °C in a vacuum oven for 12 h.

### Preparation of the CoNiFe-LDH/CNFs-0.5 composite

The CoNiFe-LDH/CNFs-0.5 composite was prepared via an easy *in situ* growth approach. Firstly, the as-prepared acid-treated CNFs were dispersed in deionised water with the assistance of sonication for 20 min to form a homogeneous CNFs dispersion (0.5 mg mL^−1^). Then, CoCl_2_·6H_2_O (0.0947 g), NiCl_2_·6H_2_O (0.5706 g) and FeCl_3_ · 6H_2_O (0.2164 g) were added into the above CNFs suspension (80 mL). After ultrasonic treatment for another 20 min, urea (0.4805 g) and sodium citrate (0.0135 g) were added under stirring. Then, the solution was transferred into a Teflon-lined autoclave. The autoclave was sealed after purging with nitrogen gas and hydrothermally treated at 150 °C for 48 h. Finally, the resulting product was filtered, washed with deionised water and alcohol until pH = 7 and dried at 80 °C for 12 h in a vacuum oven. The concentration of the CNFs was adjusted to 0.25 and 0.75 mg mL^−1^ and the corresponding composites were labeled as CoNiFe-LDH/CNFs-0.25 and CoNiFe-LDH/CNFs-0.75, respectively.

For comparison, pure CoNiFe-LDH was also prepared using the same procedure with CoCl_2_·6H_2_O, NiCl_2_ · 6H_2_O, FeCl_3_, urea and sodium citrate but without CNFs.

### Structural characterization

X-ray diffraction (XRD) analysis was carried out with a D/Max2550VB+/PC X-ray diffractometer with Cu Kα (λ = 0.15406 nm), using an operation voltage and current of 40 kV and 30 mA, respectively. TEM images were collected using a JEM-2100 microscope working at 200 kV. Specimens for observation were prepared by dispersing the samples into alcohol by ultrasonic treatment and dropped on carbon-copper grids. SEM and EDS images were collected using a Zeiss Merlin microscope working at 15 kV. The composition of the products was determined by X-ray photoelectron spectroscopy (XPS, Thermo ESCALAB 250). A Beckman coulter-type nitrogen adsorption-desorption apparatus (ASAP 2020 M) was used to investigate the pore property degassing at 120 °C for 12 h below 10^−3^ mmHg. The pore size distribution was calculated by the BJH (Barrett-Joyner-Halenda) method from the desorption branch.

### Preparation of the electrode

The working electrodes were prepared by mixing the as-prepared active material (80 wt.%), acetylene black (10 wt.%) and polyvinylidene fluoride (10 wt.%) with small amounts of ethanol to obtain a uniform black paste^3^. Then, the mixture was coated onto Ni foam and dried at 80 °C for 12 h in a vacuum.

### Electrochemical measurements

The electrochemical measurements of as-synthesized hybrids were carried out in a standard three-electrode cell at ambient temperature. Cyclic voltammetry (CV), galvanostatic charge-discharge (GCD) measurements and electrochemical impedance spectroscopy (EIS) were conducted in an electrochemical working station (CHI 660E, Chenhua, Shanghai). CV and GCD were recorded in a potential window between 0–0.6 V and 0–0.4 V, respectively. EIS was measured in the frequency range from 100 kHz to 0.01 Hz at the open circuit voltage with a 5 mV disturbance signal. Cycle life was conducted on a LAND CT2001A test system by GCD techniques. The electrochemical performance of samples was investigated in 6 M KOH aqueous solution. A platinum foil (4 cm^2^) and Hg/HgO electrode were used as counter and reference electrodes, respectively. The specific capacitance (*C*_*s*_) of the electrode materials was calculated using the following equation (1):

1 $${C}_{S}=\frac{I\times t}{{\rm{\Delta }}V\times m}$$

where I is the constant current (A), *t* is the discharge time (s), *ΔV* is the potential window of discharge (V) and *m* is the mass of active materials (g).

### Asymmetric supercapacitor

To analyze the capacitive efficiency of the CoNiFe-LDH/CNFs-0.5 electrode in a full-cell configuration, The AC//CoNiFe-LDH/CNFs-0.5 ASC was also fabricated. AC, the CoNiFe-LDH/CNFs-0.5 composite and 6 M KOH solution was used as negative electrode, positive electrode and electrolyte, respectively. The negative electrode was also prepared based on the above mentioned procedure. The mass ratio of the negative electrode to the positive electrode was decided based on charge balance theory^11^. The charge stored (*q*) by each electrode depended on the following equation (2):

2 $$q=C\times {\rm{\Delta }}V\times m$$

where *C* is specific capacitance (F g^−1^), *ΔV* is the potential window (V), *m* is the mass of active material (g).

To obtain q_+_ = q_−_, the mass balancing follows the equation (3):

3 $$\frac{{m}_{+}}{{m}_{-}}=\frac{{C}_{-}\times {\rm{\Delta }}{V}_{-}}{{C}_{+}\times {\rm{\Delta }}{V}_{+}}$$

where *m*_+_ and *m*_−_ is the mass of active material, positive and negative electrode materials, *C*_+_ and *C*_−_ is the specific capacitance of the positive and negative electrodes, *ΔV*_+_ and *ΔV*_−_ is the potential window of the positive and negative electrodes and *t* is the discharge time. The optimum weight ratio between the positive and negative electrode is calculated to be *m*_+_/*m*_−_ ≈ 0.3.

The power density (*P*) and energy density (*E*) of the device were calculated based on the following equations (4) and (5):

4 $$E=\frac{1}{2}C{\rm{\Delta }}{V}^{2}$$

5 $$P=\frac{E\times 3600}{t}$$

where *C* is specific capacitance (F g^−1^), *ΔV* is the potential window (V) and *t* is the discharge time (s).

## Summary

In summary, we developed a novel CoNiFe-LDH/CNFs-0.5 composite through an easy *in situ* growth approach for supercapacitors. The as-prepared CoNiFe-LDH/CNFs-0.5 composite exhibited higher specific surface area (114.2 m^2^ g^−1^), specific capacitance (1203 F g^−1^ at 1 A g^−1^) and rate capability (77.1% from 1 A g^−1^ to 10 A g^−1^) than pure CoNiFe-LDH. After 1000 cycles, the capacitive retention rate of the CoNiFe-LDH/CNFs-0.5 composite remained at 94.4%, demonstrating excellent long-time cycle stability. The AC//CoNiFe-LDH/CNFs-0.5 ASC based on CoNiFe-LDH/CNFs-0.5 as a positive electrode and AC as a negative electrode was manufactured and it exhibited a specific capacitance of 84.9 F g^−1^ at 1 A g^−1^ and a high energy density of 30.2 W h kg^−1^. More importantly, this device showed long-term cycling stability, with 82.7% capacity retention after 2000 cycles. This impressive electrochemical performance of the CoNiFe-LDH/CNFs-0.5 composite indicated significant potential for application in high-power and energy-storage devices.

## Electronic supplementary material

Supplementary information

## References

[1] Huang Y, Miao Y-E, Tjiu WW, Liu T (2015). High-performance flexible supercapacitors based on mesoporous carbon nanofibers/Co_3_O_4_/MnO_2_ hybrid electrodes. RSC Adv..

[2] Xu Y (2015). General Strategy to Fabricate Ternary Metal Nitride/Carbon Nanofibers for Supercapacitors. Chem. Electro. Chem..

[3] He F (2014). *In situ* fabrication of nickel aluminum-layered double hydroxide nanosheets/hollow carbon nanofibers composite as a novel electrode material for supercapacitors. J. Power Sources.

[4] Zhang Z, Xiao F, Xiao J, Wang S (2015). Functionalized carbonaceous fibers for high performance flexible all-solid-state asymmetric supercapacitors. J. Mater. Chem. A.

[5] Zhao J (2013). Flexible hierarchical nanocomposites based on MnO_2_ nanowires/CoAl hydrotalcite/carbon fibers for high-performance supercapacitors. RSC Adv..

[6] Zhang W (2013). Asymmetric electrochemical capacitors with high energy and power density based on graphene/CoAl-LDH and activated carbon electrodes. RSC Adv..

[7] Tang S, Zhu B, Shi X, Wu J, Meng X (2017). General Controlled Sulfidation toward Achieving Novel Nanosheet-Built Porous Square-FeCo_2_S_4_-Tube Arrays for High-Performance Asymmetric All-Solid-State Pseudocapacitors. Adv. Energy Mater..

[8] Yang P (2014). Low-cost high-performance solid-state asymmetric supercapacitors based on MnO_2_ nanowires and Fe_2_O_3_ nanotubes. Nano letters.

[9] Zhou M (2015). One-step route synthesis of active carbon@La_2_NiO_4_/NiO hybrid coatings as supercapacitor electrode materials: Significant improvements in electrochemical performance. J. Electroanal. Chem..

[10] Liu M (2015). Eco-friendly synthesis of hierarchical ginkgo-derived carbon nanoparticles/NiAl-layered double hydroxide hybrid electrodes toward high-performance supercapacitors. RSC Adv..

[11] Chen Z (2011). High-performance supercapacitors based on intertwined CNT/V_2_O_5_ nanowire nanocomposites. Adv. Mater..

[12] Kumar R, Rai P, Sharma A (2016). 3D urchin-shaped Ni_3_(VO_4_)_2_ hollow nanospheres for high-performance asymmetric supercapacitor applications. J. Mater. Chem. A.

[13] Xie L-j (2016). A high energy density asymmetric supercapacitor based on a CoNi-layered double hydroxide and activated carbon. New Carbon Mater..

[14] Zhou P (2016). Facile hydrothermal synthesis of NiTe and its application as positive electrode material for asymmetric supercapacitor. J. Alloy Compd..

[15] Sawangphruk M (2013). High-performance supercapacitors based on silver nanoparticle-polyaniline-graphene nanocomposites coated on flexible carbon fiber paper. J. Mater. Chem. A.

[16] Shi S, Zhuang X, Cheng B, Wang X (2013). Solution blowing of ZnO nanoflake-encapsulated carbon nanofibers as electrodes for supercapacitors. J. Mater. Chem. A.

[17] Li Y (2015). Nitrogen-and oxygen-enriched 3D hierarchical porous carbon fibers: synthesis and superior supercapacity. J. Mater. Chem. A.

[18] Dong Y (2015). Synthesis of mesoporous graphitic carbon fibers with high performance for supercapacitor. Electrochim. Acta.

[19] Yu D (2015). Transforming Pristine Carbon Fiber Tows into High Performance Solid-State Fiber Supercapacitors. Adv. Mater..

[20] Das A, Schutzius TM, Bayer IS, Megaridis CM (2012). Superoleophobic and conductive carbon nanofiber/fluoropolymer composite films. Carbon.

[21] Jagadale AD (2016). Binder-Free Electrodes of CoAl Layered Double Hydroxide on Carbon Fibers for All-Solid-State Flexible Yarn Supercapacitors. Energy Technol..

[22] Ardanuy M, Rodríguez-Perez MA, Algaba I (2011). Electrical conductivity and mechanical properties of vapor-grown carbon nanofibers/trifunctional epoxy composites prepared by direct mixing. Compos. Part B: Eng..

[23] Ge J (2016). Elastic and hierarchical porous carbon nanofibrous membranes incorporated with NiFe_2_O_4_ nanocrystals for highly efficient capacitive energy storage. Nanoscale.

[24] Liu X (2014). A NiAl layered double hydroxide@carbon nanoparticles hybrid electrode for high-performance asymmetric supercapacitors. J. Mater. Chem. A.

[25] Heli H, Pishahang J, Amiri HB (2016). Synthesis of hexagonal CoAl-layered double hydroxide nanoshales/carbon nanotubes composite for the non-enzymatic detection of hydrogen peroxide. J. Electroanal. Chem..

[26] Zhao J (2013). CoMn-layered double hydroxide nanowalls supported on carbon fibers for high-performance flexible energy storage devices. J. Mater. Chem. A.

[27] Chen J, Xu J, Zhou S, Zhao N, Wong C-P (2016). Amorphous nanostructured FeOOH and Co-Ni double hydroxides for high-performance aqueous asymmetric supercapacitors. Nano Energy.

[28] Chen S (2016). A high-rate cathode material hybridized by in-site grown Ni-Fe layered double hydroxides and carbon black nanoparticles. J. Mater. Chem. A.

[29] Yang J (2013). Facile fabrication of MWCNT-doped NiCoAl-layered double hydroxide nanosheets with enhanced electrochemical performances. J. Mater. Chem. A.

[30] Yu C (2014). Nanohybrids from NiCoAl-LDH coupled with carbon for pseudocapacitors: understanding the role of nano-structured carbon. Nanoscale.

[31] Yang J, Yu C, Fan X, Qiu J (2014). 3D Architecture Materials Made of NiCoAl-LDH Nanoplates Coupled with NiCo-Carbonate Hydroxide Nanowires Grown on Flexible Graphite Paper for Asymmetric Supercapacitors. Adv. Energy Mater..

[32] Ma X, Li H, Zhu G, Kang L, Liu Z-H (2010). Hydrothermal preparation and anion exchange of Co^2+^-Ni^2+^-Fe^3+^ CO_3_^2−^ LDHs materials with well regular shape. Colloids and Surfaces A: Physicochem. Eng. Aspects.

[33] Li M, Cheng JP, Wang J, Liu F, Zhang XB (2016). The growth of nickel-manganese and cobalt-manganese layered double hydroxides on reduced graphene oxide for supercapacitor. Electrochim. Acta.

[34] Li H, Zhu G, Liu Z-H, Yang Z, Wang Z (2010). Fabrication of a hybrid graphene/layered double hydroxide material. Carbon.

[35] Cai X (2015). Solvothermal synthesis of NiCo-layered double hydroxide nanosheets decorated on RGO sheets for high performance supercapacitor. Chem. Eng. J.

[36] Bilovol V, Ferrari S, Derewnicka D, Saccone FD (2014). XANES and XPS study of electronic structure of Ti-enriched Nd-Fe-B ribbons. Mater. Chem. Phys..

[37] Yang W (2013). Solvothermal one-step synthesis of Ni-Al layered double hydroxide/carbon nanotube/reduced graphene oxide sheet ternary nanocomposite with ultrahigh capacitance for supercapacitors. ACS Appl. Mater. Interfaces.

[38] Shao M (2012). Core-Shell Layered Double Hydroxide Microspheres with Tunable Interior Architecture for Supercapacitors. Chem.Mater..

[39] Zhang L (2012). Enhanced high-current capacitive behavior of graphene/CoAl-layered double hydroxide composites as electrode material for supercapacitors. J. Power Sources.

[40] Gao Z (2011). Graphene Nanosheet/Ni^2+^/Al^3+^Layered Double-Hydroxide Composite as a Novel Electrode for a Supercapacitor. Chem. Mater..

[41] Yan J (2010). Rapid microwave-assisted synthesis of graphene nanosheet/Co_3_O_4_ composite for supercapacitors. Electrochim. Acta.

[42] Warsi MF (2014). Conformal Coating of Cobalt-Nickel Layered Double Hydroxides Nanoflakes on Carbon Fibers for High-performance Electrochemical Energy Storage Supercapacitor Devices. Electrochim. Acta.

[43] Yang B, Yang Z, Wang R, Wang T (2013). Layered double hydroxide/carbon nanotubes composite as a high performance anode material for Ni-Zn secondary batteries. Electrochim. Acta.

[44] Liu G, Wang L, Wang B, Gao T, Wang D (2015). A reduced graphene oxide modified metallic cobalt composite with superior electrochemical performance for supercapacitors. RSC Adv..

[45] Cheng JP (2013). Enhanced electrochemical performance of CoAl-layered double hydroxide nanosheet arrays coated by platinum films. Electrochim. Acta.

[46] Zang J (2008). Well-Aligned Cone-Shaped Nanostructure of Polypyrrole/RuO_2_ and Its Electrochemical Supercapacitor. J. Phys. Chem. C.

[47] Li Z (2016). A flexible all-solid-state micro-supercapacitor based on hierarchical CuO@layered double hydroxide core-shell nanoarrays. Nano Energy.

[48] Gu Y (2013). Preparation and capacitance behavior of manganese oxide hollow structures with different morphologies via template-engaged redox etching. J. Power Sources.

[49] Li J, Xiong S, Liu Y, Ju Z, Qian Y (2013). High electrochemical performance of monodisperse NiCo_2_O_4_ mesoporous microspheres as an anode material for Li-ion batteries. ACS Appl. Mater. Interfaces.

[50] Xu J (2014). Reduced graphene oxide/Ni_(1−x)_Co_(x)_Al-layered double hydroxide composites: preparation and high supercapacitor performance. Dalton Trans..

[51] Zhang L, Hui KN, San Hui K, Lee H (2016). High-performance hybrid supercapacitor with 3D hierarchical porous flower-like layered double hydroxide grown on nickel foam as binder-free electrode. J. Power Sources.

[52] Wang H (2012). Graphene-nickel cobaltite nanocomposite asymmetrical supercapacitor with commercial level mass loading. Nano Res..

[53] Wang X, Liu WS, Lu X, Lee PS (2012). Dodecyl sulfate-induced fast faradic process in nickel cobalt oxide-reduced graphite oxide composite material and its application for asymmetric supercapacitor device. J. Mater. Chem..

[54] Tang C, Tang Z, Gong H (2012). Hierarchically Porous Ni-Co Oxide for High Reversibility Asymmetric Full-Cell Supercapacitors. J. Electrochem. Soc..

[55] Lu XF (2014). Hierarchical NiCo_2_O_4_ nanosheets@hollow microrod arrays for high-performance asymmetric supercapacitors. J. Mater. Chem. A.

[56] Wang X, Sumboja A, Lin M, Yan J, Lee PS (2012). Enhancing electrochemical reaction sites in nickel-cobalt layered double hydroxides on zinc tin oxide nanowires: a hybrid material for an asymmetric supercapacitor device. Nanoscale.

[57] Xu K (2014). Hierarchical mesoporous NiCo_2_O_4_@MnO_2_ core-shell nanowire arrays on nickel foam for aqueous asymmetric supercapacitors. J. Mater. Chem. A.

