# Supplementary material for: High performance asymmetric supercapacitor based on Cobalt Nickle Iron-layered double hydroxide/carbon nanofibres and activated carbon
Source: Sci Rep. 2017 Jul 5;7:4707. doi: 10.1038/s41598-017-04807-1 (PMC5498571; doi:10.1038/s41598-017-04807-1)
Supplement: Supplementary file 1 — Supplementary information [file 41598_2017_4807_MOESM1_ESM.pdf]

Supplementary Information

High performance asymmetric supercapacitor based on Cobalt Nickle Iron-layered double hydroxide/carbon nanofibres and activated carbon

Feifei Wang, Shiguo Sun, Yongqian Xu, Ting Wang, Ruijin Yu and Hongjuan Li*

Shaanxi Key Laboratory of Natural Products & Chemical Biology, School of Chemistry & Pharmacy, Northwest A&F University, Xinong Road 22, Yangling, Shaanxi 712100, P. R. China

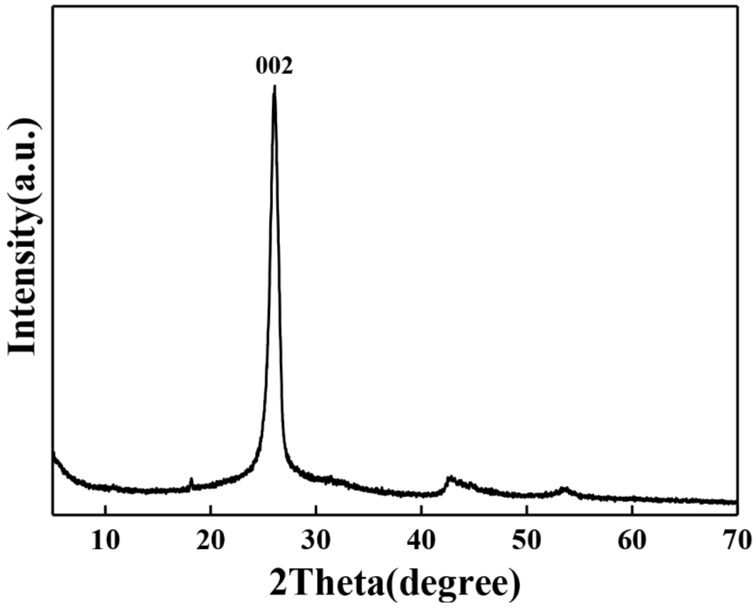

Fig S1. XRD pattern of CNFs.

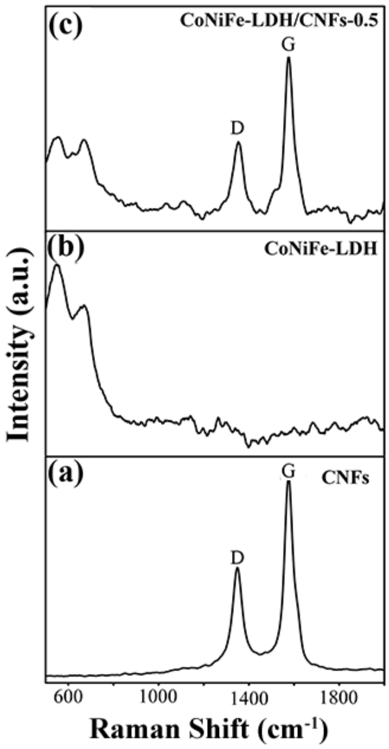

Fig S2 (a) Raman patterns of (a) CNFs, (b) CoNiFe-LDH and (c) the CoNiFe-LDH/CNFs-0.5 composite.

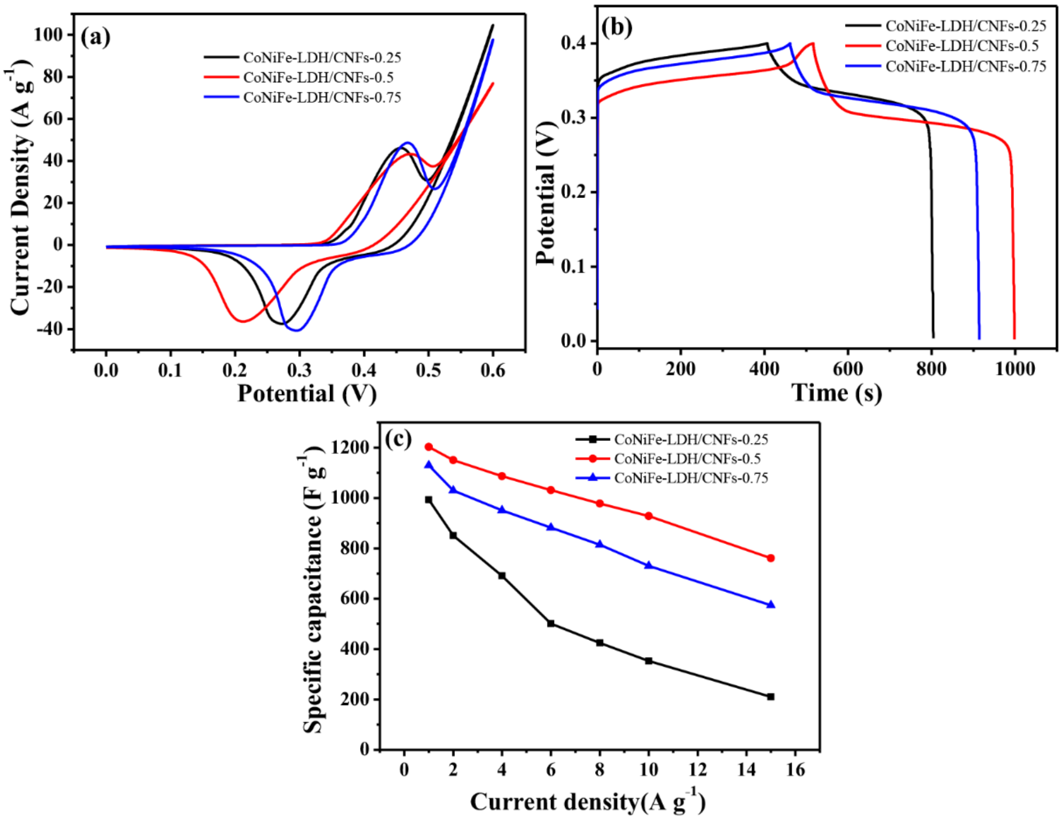

Fig S3 (a) CV curves of the CoNiFe-LDH/CNFs composites at a scan rate of 10 mV s^{-1} with different concentrations of CNFs; (b) Galvanostatic charge/discharge curves of the

CoNiFe-LDH/CNFs composites at a current density of 1 A g^{-1} with different concentrations of CNFs; (c) The specific capacitance of the CoNiFe-LDH/CNFs composites at different current densities with different concentrations of CNFs.

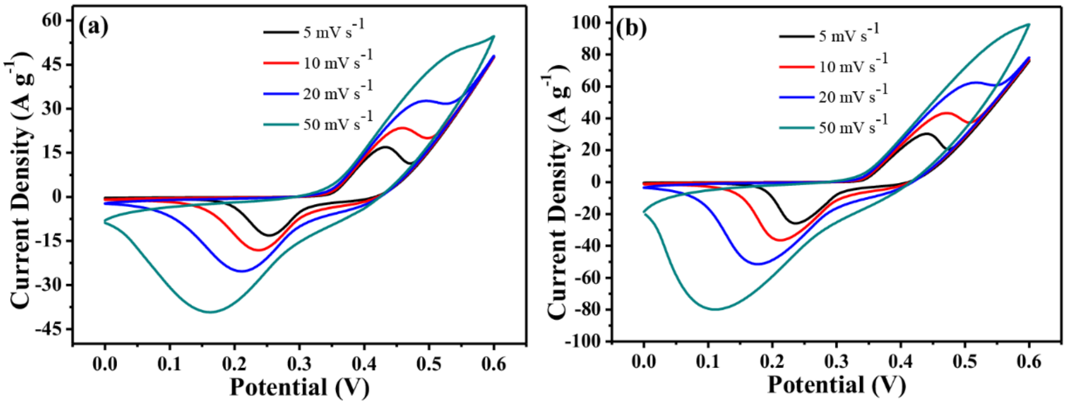

Fig. S4 (a) CV curves of the CoNiFe-LDH at different scan rates. (b) CV curves of the CoNiFe-LDH/CNFs-0.5 composite at different scan rates.

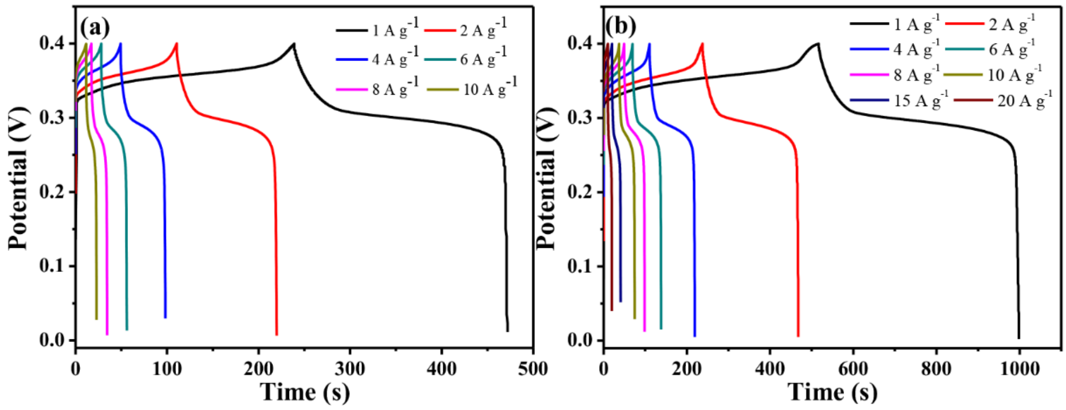

Fig. S5 (a) Galvanostatic charge/discharge curves of the CoNiFe-LDH at different current densities. (b) Galvanostatic charge/discharge curves of the CoNiFe-LDH/CNFs-0.5 composite at different current densities.
